# Research progress on the mechanism of tumor cell ferroptosis regulation by epigenetics

**DOI:** 10.1080/15592294.2025.2500949

**Published:** 2025-05-06

**Authors:** Yuyang Xiao, Mengyang He, Xupeng Zhang, Meng Yang, Zhangchi Yuan, Shanhu Yao, Yuexiang Qin

**Affiliations:** a Department of Health Management Medical, The Third Xiangya Hospital of Central South University, Changsha, Hunan, China; b Xiangya School of Medicine, Central South University, Changsha, Hunan, China; c Department of Radiology, The Third Xiangya Hospital, Central South University, Changsha, Hunan, China; d Key Laboratory of Medical Information Research, Central South University, Changsha, Hunan, China; e Department of Otolaryngology, Head and Neck Surgery, Xiangya Hospital, Central South University, Changsha, Hunan, China

**Keywords:** Ferroptosis, epigenetic regulation, cancer therapy, tumor development, regulated cell death

## Abstract

Cancer remains a significant barrier to human longevity and a leading cause of mortality worldwide. Despite advancements in cancer therapies, challenges such as cellular toxicity and drug resistance to chemotherapy persist. Regulated cell death (RCD), once regarded as a passive process, is now recognized as a programmed mechanism with distinct biochemical and morphological characteristics, thereby presenting new therapeutic opportunities. Ferroptosis, a novel form of RCD characterized by iron-dependent lipid peroxidation and unique mitochondrial damage, differs from apoptosis, autophagy, and necroptosis. It is driven by reactive oxygen species (ROS)-induced lipid peroxidation and is implicated in tumorigenesis, anti-tumor immunity, and resistance, particularly in tumors undergoing epithelial-mesenchymal transition. Moreover, ferroptosis is associated with ischemic organ damage, degenerative diseases, and aging, regulated by various cellular metabolic processes, including redox balance, iron metabolism, and amino acid, lipid, and glucose metabolism. This review focuses on the role of epigenetic factors in tumor ferroptosis, exploring their mechanisms and potential applications in cancer therapy. It synthesizes current knowledge to provide a comprehensive understanding of epigenetic regulation in tumor cell ferroptosis, offering insights for future research and clinical applications.

## Introduction

**Figure 1.**
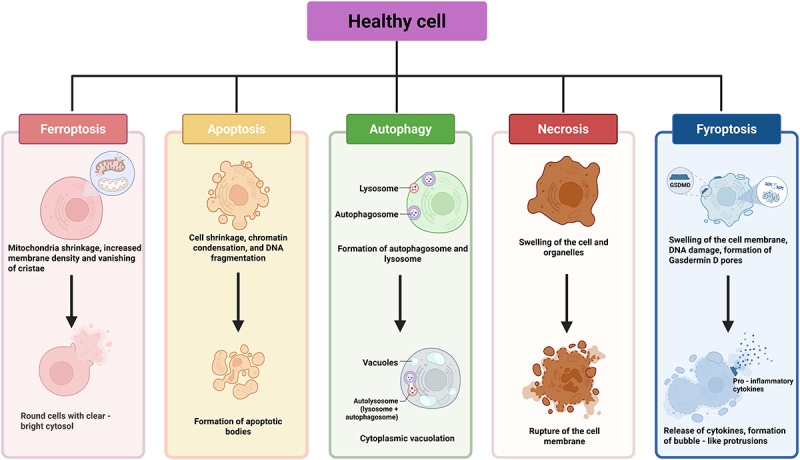
Schematic diagram of the feature comparisons between ferroptosis and other cell death modes.

Ferroptosis is a distinct form of regulated cell death (RCD), first introduced by B.R. Stockwell in 2012 [1]. It is characterized by the excessive intracellular accumulation of iron ions and reactive oxygen species (ROS), which trigger lipid peroxidation and ultimately lead to cell death [1,2] (Figure 1). The defense mechanisms for ferroptosis are cellular antioxidant systems that balance lipid peroxidation, each with a unique subcellular localization and complex regulatory mechanisms, including SLC7A11–GSH – GPX4 defense system, FSP1-CoQH2 defense system, GCH1-BH4 defense system, DHODH-CoQH2 defense system and Thioredoxin antioxidant system. Ferroptosis can be regulated by directly or indirectly inhibiting lipid peroxidation or by pharmacologically reducing intracellular iron levels through epigenetic mechanisms [1,3]. Additionally, it is influenced by various pathways, including redox homeostasis, iron metabolism, mitochondrial stability, and the metabolism of amino acids, lipids, and glucose [4], thereby playing a crucial role in cellular functions. Research indicates that ferroptosis is significantly involved in tumor development, anti-tumor immunity, tumor resistance, and other related processes [5–7], as well as in ischemic organ damage, degenerative diseases, and cellular senescence [8], highlighting its substantial research significance.

Numerous studies have demonstrated that ferroptosis is precisely regulated by protein post-translational modifications (PTMs) and epigenetic modifications [3,9–12]. Epigenetic modifications refer to heritable changes in gene activity that occur through covalent modifications of nucleic acids and histones, collectively regulating gene function, expression, and chromatin structure [13,14] without altering the underlying DNA sequence [15]. These modifications are reversible and dynamically regulated, occurring at various levels of gene expression, including DNA methylation, histone PTMs, chromatin remodeling (the dynamic spatiotemporal localization of nucleosomes), and non-coding RNA (ncRNA) regulation [13,14]. Epigenetic mechanisms comprise various enzymes and protein domains [16] that fine-tune gene expression programs, thereby controlling essential biological processes such as cell differentiation and embryogenesis [17].

Cancer poses one of the greatest threats to global public health, significantly impacting human health and socio-economic development [18–20]. According to WHO estimates, approximately 20 million new cases of malignant tumors and 9.7 million related deaths occurred worldwide in 2022, accounting for two-thirds of new cases and deaths globally. It is projected that the number of new cases will reach 35 million by 2025, representing a 77% increase over 2022. Understanding the mechanisms underlying cancer occurrence and development, as well as researching cancer prevention, diagnosis, and treatment, remains a primary focus of medical research. Currently, cancer treatment primarily involves surgical resection, radiotherapy, and chemotherapy [21]; however, these conventional methods carry risks of recurrence and metastasis and often have significant side effects. Therefore, exploring novel cancer treatment strategies is urgent. Research has shown that uncontrolled ferroptosis plays a critical role in tumor development, closely linked to the regulation of epigenetics on ferroptosis in tumor cells, thus providing a new avenue for tumor treatment [3].

This review summarizes the molecular mechanisms of ferroptosis and then discusses the role of epigenetic regulation of ferroptosis in tumor development. Finally, we explore the potential applications of targeted ferroptosis epigenetic regulators in tumor therapy and anticipate future advancements in this field.

## Molecular mechanisms of ferroptosis

### Main characteristics of ferroptosis

Compared with other modes of cell death such as apoptosis, necrosis, and autophagy, ferroptosis is morphologically, biochemically, immunologically unique [1,22]. Each of these aspects is discussed below.

#### Morphological characteristics of ferroptosis

Cells undergoing ferroptosis show a series of specific morphological changes, including loss of cytoplasmic membrane integrity and swelling of the cytoplasm and organelles [23], but the nucleus remains intact, with no chromatin condensation or margination. Ferroptosis under certain conditions is accompanied by cell detachment and aggregation, with a corresponding increase in the number of autophagic vesicles [24]. More uniquely, cellular ferroptosis can rapidly propagate along intercellular contacts in the form of waves that spread to neighboring cells [25], but the mechanism remains unclear. At the ultrastructural level, iron-dead cells usually exhibit mitochondrial abnormalities, including mitochondrial condensation, decreased number, reduced volume, increased density of the bilayer membrane, decreased or absent mitochondrial cristae, and rupture of the outer membrane [26]. It has been shown that mitochondria-mediated ROS production, DNA stress and metabolic reprogramming are required for the induction of lipid peroxidation and ferroptosis [23,27].

#### Biochemical characterization of ferroptosis

Iron ion metabolism has a key role in ferroptosis, lipid peroxidation initiation can be hindered by iron chelators, and Fe^2+^ supplementation increases cellular sensitivity to ferroptosis inducers [1]. The accumulation of intracellular free iron ions directly triggers cellular ferroptosis, along with excess ferrous and nonferrous heme iron [24,28]. Overaccumulation of Fe^2+^ correlates significantly with ROS overload and lipid peroxidation [29], Fe^2+^ oxidizes lipids through the Fenton reaction and causes a cascade reaction occurs, leading to significant ROS accumulation, and when intracellular reduced glutathione (GSH) is depleted or glutathione peroxidase 4 (GPX4) activity is reduced, ROS are not scavenged in a timely manner, and the cell will undergo ferroptosis [30]. And then the cellular antioxidant defense system is suppressed, oxidative stress is exacerbated, and intracellular antioxidant enzymes suffer, further deteriorating the oxidative state of the cells [31].

Lipid metabolism during ferroptosis is also significantly characterized. During ferroptosis, the concentration of reactive aldehydes such as malondialdehyde (MDA) and 4-hydroxynonenal (4HNE), which are important products of lipid peroxidation, continues to rise [32]. Furthermore, peroxidation of polyunsaturated fatty acids (PUFAs) plays a key role in ferroptosis [32]. Notably, lipid peroxidation in ferroptosis is not an isolated event; it is closely related to other intracellular biochemical reactions, which together lead to impairment of cellular structure and function and drive the ferroptosis process [33].

#### Immunological characterization of ferroptosis

The effects of ferroptosis on immunity are manifold. Ferroptosis damages leukocytes and affects immune function; lipid peroxidation induces ferroptosis in T cells and exacerbates viral and parasitic infections [34]. In addition, ferroptosis alters the immune system’s handling of antigens released by dead cells and the corresponding antibody production [35]. In ferroptosis, 4HNE regulates the expression of a variety of genes associated with immune and inflammatory responses through the activation of transcription factors such as NF-κB [36], which affects cell signaling and gene expression and regulates immune and inflammatory responses [37]. Ferroptosis may also interfere with the clearance of dead cells, affect the release and activation of damage-associated molecular patterns (DAMP), and exacerbate immune, inflammatory, and autoimmune responses [34,38], affecting an individual’s health and disease state.

### Ferroptosis inducers and inhibitors

Four classes of ferroptosis inducers have been identified, Erastin, RSL3 and its analogs ML162 and ML210, FIN56 and FINO2, which induce ferroptosis in different ways. Inhibitors of ferroptosis are the oxidizing inhibitor Ferrostatin-1 (Fer-1), Deferoxamine (DFO) and water soluble Vitamin E. The targets and mechanisms by which the above substances induce and inhibit ferroptosis are detailed in Table 1. With in-depth studies on the mechanisms of ferroptosis, their inducers and inhibitors will provide new ideas for the treatment of many diseases [1].

**Table 1.** Ferroptosis inducers and inhibitors.

Reagents	Classification	Targets	Mechanism of action	References
Ferroptosis Inducers	Erastin	SLC7A11	Reduces GSH levels by inhibiting SLC7A11 thereby inducing cellular ferroptosis.	Dixon et al. [1]
	Erastin	VDAC2, VDAC3	Targeting VDAC2 and VDAC3 leads to cell death by inducing mitochondrial dysfunction.	Dixon et al. [1]
	Erastin	–	Erastin accumulation in the endoplasmic reticulum suggests that the endoplasmic reticulum may also be one of the key target sites for Erastin-induced ferroptosis to occur.	Gaschler et al. [39]
		–	It has been found that lipid hydroperoxides are mainly concentrated in the endoplasmic reticulum, which corroborates the existence of a complex mechanism for Erastin-induced ferroptosis.	Kagan et al. [40]
	RSL3, ML162, ML210	GPX4	Inhibition of GPX4 activity in cells and mitochondria increases lipid ROS and lipid peroxidation.	Yang and Stockwell [41], Weïwer et al. [42]
	FIN56	–	Directly promotes the degradation of GPX4.	Shimada et al. [43]
	FIN56	–	Binding to squalene synthase leads to depletion of endogenous antioxidant coenzyme Q10 and enhances cellular sensitivity to FIN56-induced ferroptosis.	Shimada et al.[43]
	FINO2	–	Induces mitochondrial lipid peroxidation and oxidized Fe^2+^ production, which leads to ferroptosis due to the combined effects of direct oxidation of unstable iron and GPX4 inactivation.	Abrams et al. [44], Gaschler et al. [45]
	FAC	–	Promotes iron overload via Fe^3+^ accumulation, enhancing Fenton reactions and lipid peroxidation.	Battaglia et al. [46]
	Ferlixit	–	Provides exogenous Fe^3+^, increases intracellular LIP, and induces ferroptosis in cells.	Battaglia et al.[46]
Ferroptosis Inhibitors	Fer-1	ROS	Remove oxides.	Dixon et al. [1]
	DFO	ROS	Remove oxides.	Dixon et al. [1]
	Vitamin E	ROS	Remove oxides.	Dixon et al. [1]

### Mechanisms of non-epigenetic regulation of ferroptosis

Four major mechanisms of non-epigenetic regulation of ferroptosis play an important role in ferroptosis, including iron ion metabolism, lipid metabolism, oxygen-containing radicals, and the degradation system.The four mechanisms of non-epigenetic regulation of ferroptosis are listed in Table 2. Exploring the intricate mechanisms underlying ferroptosis reveals novel therapeutic perspectives for the treatment of a spectrum of diseases.

**Table 2.** Mechanisms of non-epigenetic regulation of ferroptosis.

Methods of regulation	Interacting molecule	Target of action	Effect of action	References
Ferrous ion metabolism	Ferrous ion chelator	–	Increase in intracellular unstable ferrous ions during the process, effectively inhibiting the onset of ferroptosis.	Dixon et al. [1], Holliday [15]
	Ferrous ion	Electron transport chain, redox metabolism, and DNA synthesis	Affects cellular sensitivity to ferroptosis. Abnormalities in iron intake, storage, utilization, and metabolism may lead to pathological accumulation of iron and enhance cellular lipid peroxidation damage due to the Fenton reaction.	Dixon et al. [1], Friedmann Angeli and Conrad [47]
	TF	Non-heme iron	Uptake of exogenous iron ions. TF dissociates Fe^3+^ at low pH in vesicles. Iron ion uptake and transport are inhibited in the absence of TF function, as is ferroptosis.	Alim et al. [48], Bersuker et al. [49]
	TFRC	TF incorporating non-heme iron	TFs that bind nonheme iron are recognized in the cell membrane and cytosolized into the cell, forming specialized vesicles. Iron ion uptake and transport are inhibited in the absence of TFRC function, as is ferroptosis.	Alim et al. [48], Bersuker et al. [49]
	STEAP3	Fe^3+^	Fe^3+^ is reduced to Fe^2+^ by the iron reductase STEAP3.	Alim et al. [48]
	SLC11A2/DMT1	Fe^2+^	Transport of Fe^2+^ into the cytoplasm is involved in ferroptosis.	Bersuker et al. [49]
	LIP	Fe^2+^	Plays a transit role in the storage, transport and utilization of iron ions. Enriched for Fe^2+^, which is normally translocated into mitochondria for the synthesis of ferrous heme and iron-sulfur clusters.	Doll et al. [50], Stefely and Pagliarini [51]
	SLC25, Mitochondrial ferritin	Iron in the cytoplasm	Translocated into the mitochondrial membrane.	Fanet et al.[52], Kraft et al. [53]
	PINK1/PRKN/PARK2	SLC25	Degradation of SLC25, limiting iron ion accumulation and inhibiting mitochondrial pathway ferroptosis.	Fanet et al. [52], Kraft et al. [53]
	SLC40A1	Fe^2+^	Translocates Fe^2+^, a process that requires extracellular oxidation of Fe^2+^ to Fe^3+^.	Soula et al. [54]
	CP	Iron in SLC40A1	Oxidation of iron in SLC40A1, negatively regulates hepatocyte ferroptosis, and CP deficiency can increase intracellular Fe^2+^ and lipid peroxidation.	Soula et al. [54]
	LOX, POR	Ferrous ion	Iron ions are important cofactors for LOX and POR, both of which play key roles in ferroptosis in ferroptosis.In essence, disruptions in intracellular iron homeostasis, particularly Fe^2+^ accumulation, can provoke ferroptosis. Lowering intracellular Fe^2+^ concentrations effectively thwarts ferroptosis.	Mao et al. [55]
Lipid metabolism	LOX, POR	–	Produces lipid peroxides, and its characteristic diffusion pattern relies on LIP-mediated diffusion of peroxides across the membrane.	Ding et al. [56]
	ACSL4, LPCAT3	PUFAs	ACSL4 catalyzes the biochemical reaction of AA and AdA adrenate with CoA to generate AA/AdA-CoA derivatives esterified to phospholipids. LPCAT3 catalyzes the biosynthesis of AA/AdA-CoA with membrane PE to generate AA/AdA-PEPUFA.PUFA promotes lipid peroxidation.	Lee et al. [57], Ma et al. [58]
	FSP1	CoQ10	Catalyses the regeneration of CoQ10 using NAD(P)H, reduces CoQ10 to CoQH2. CoQH2 can trap lipid peroxyl radicals, thereby preventing the accumulation of lipid peroxides in the cell membrane and inhibiting ferroptosis.	Doll et al. [50]
	FAO	Fatty acids	Consume most of the fatty acids, resulting in a reduced rate of lipid peroxidation.	Gao et al. [59]
	ACLY	CoA	Catalyzes the production of citric acid.	Kakhlon and Cabantchik [60]
	ACSF2, CS	Mitochondrial fatty acid metabolism	Required for mitochondrial fatty acid metabolism. Knockdown reversed ferroptosis, suggesting a role for mitochondrial fatty acid metabolism in promoting ferroptosis.	Hogg et al. [14], Philpott et al. [61]
	TOFA	PUFA	Inhibition of ferroptosis, demonstrating the key role of PUFA as a substrate for ferroptosis lipid peroxidation.	Wu et al. [3], Paradkar et al. [62]
	MUFAs	–	It is not susceptible to peroxidation and inhibits ferroptosis.	Li et al. [63]
	ACSL3, SCD1	MUFA	Catalyzes MUFA synthesis. Also has an inhibitory effect on ferroptosis.	Shang et al. [64]
Oxygen-containing radicals	NOX	ROS	ROS generation primarily depends on mitochondrial metabolism and the activity of NOX located in the cell membrane.	Hogg et al. [14]
	AMPK	–	Maintains ATP homeostasis and regulates ROS production through protein phosphorylation.	Wu et al. [65]
	VDAC	–	Controls the exchange of substances between mitochondria and other organelles during oxidative stress.	Zou et al. [66]
	RSL3	VDAC2	VDAC2 is directly targeted for carbonylation during RSL3-induced ferroptosis.	Geng et al. [67]
	Erastin	VDAC2, VDAC3	Erastin can induce the degradation of VDAC2 and VDAC3 via an E3 ubiquitin ligase-dependent mechanism, thereby promoting ferroptosis.	Geng et al. [67]
	NOXs	Complexes composed of cell membranes and enzymes	Transport of electrons through the plasma membrane generates superoxide and other ROS, which contribute to lipid peroxidation in ferroptosis.	Kagan et al. [40], Stockwell et al. [68], Doll et al. [69], Liang et al. [70], Tesfay et al. [71]
Degradation system	Oxidative stress and lipid peroxidation products	–	A powerful inducer of autophagy, excessive autophagy enhances ferroptosis.	[32]
	Rapamycin	mTOR	Leading to the formation of autophagic vesicles.	[72,73]
	Autophagy effector	–	Inhibition of cancer cell ferroptosis.	Holliday [15], Lee et al. [72], Yang et al. [74]
	SLC7A11, GPX4	–	SLC7A11 and GPX4 deletion impairs the autophagic process and resists ferroptosis triggered by Golgi stress, revealing a complex interplay between autophagy, ferroptosis, and organelle stress.	Holliday [15], Lee et al. [72], Yang et al. [74]
	OTU deubiquitinases, OTUB1	SLC7A11	Stabilizes SLC7A11. Inhibition of OTUB1 enhances SLC7A11 degradation and sensitivity of cancer cells to ferroptosis.	Li et al. [75]
	ACSL4, VDAC2, VDAC3	–	Regulation of protein stability at the ferroptosis promoter.	Shoshan-Barmatz et al.[76]
	RNF113A	–	Plays a key role in DNA damage-induced ferroptosis. deletion of RNF113A stimulates an ferroptosis process that is tightly linked to DNA damage.	Yang et al. [77]
	LONP1	–	Mediates mitochondrial degradation, leading to mitochondrial DNA damage and activation of STING1/TMEM173, which triggers an autophagic response that leads to ferroptosis in pancreatic cancer cells.	Wu et al. [65]
	NEDD4	VDAC2, VDAC3	Inhibition of ferroptosis.	Bedard and Krause [78]
	NEDD4L	LTF	Slow down the accumulation of iron in cancer cells and inhibit ferroptosis triggered by lipid peroxidation.	Chen et al. [79]

## Epigenetic regulation of ferroptosis

Epigenetic modifications encompass histone posttranslational modifications, DNA and RNA methylation, and ncRNA regulation, etc. The roles of these modifications in regulating tumor ferroptosis are outlined below.

### Posttranslational modification of histones regulates ferroptosis

The nucleosome is the fundamental unit of chromatin, comprising a segment of DNA and an octamer of four core histones H3, H4, H2A, and H2B. These histones serve as scaffolds that encapsulate and concentrate the DNA, playing a critical role in the compaction and disassembly of chromatin [80,81]. Histone post-translational modifications (PTMs) are a group of multifunctional epigenetic marks that regulate the conformation of chromatin and the accessibility of transcription factors, co-activators and co-inhibitors as well as involve in transcription, DNA damage, apoptosis, and cell cycle regulation [80,82]. The miswriting, misinterpretation and misclearance of histone modifications are closely related to the development of tumors, and the disturbance of histone coding leads to the disorder of gene expression and cell characteristics [83,84].

Epigenetic modifications and PTMs regulate gene expression during transcription and post-transcription respectively, and regulate protein activity, function, and degradation after transcription [85]. Their dysregulation results in abnormal gene and protein expression, contributing to the transformation into malignant phenotypes and facilitating tumor initiation and progression [85–87].

#### Histone methylation regulates ferroptosis

Histone methylation modifications associated with ferroptosis mainly involve the methylation of H3K4 and H3K9, catalyzed by histone methyltransferase (HMTs) [88]. Research indicates that GPX4 is more abundantly expressed in tumor cells than in normal cells, which correlates with increased levels of H3K4me3 on the GPX4 promoter [89]. Methionine adenosine transferase 2A (MAT2A) promotes the production of methylated donor SAM, which upregulates ACSL3 by increasing the abundance of H3K4me3 on the promoter, thereby inhibiting ferroptosis [90]. JQ1 inhibits HMT expression called G9a, reducing H3K4me3 abundance of BRD4, and finally induces ferroptosis in cancer cells [91]. In breast cancer, mucin 1-C binds to the CD44 variant to stabilize the SLC7A11 molecule, while H3K9me2/3 on the Mucin 1-C promoter suppresses its transcription, thereby affecting GPX4’s capacity to induce ferroptosis [92]. Branched-chain amino acid transaminase-1 (BCAT1) can regulate cellular ferroptosis by modulating H3K9me3. The absence of H3K9me3 at the EGR1 promoter site leads to rapid upregulation of EGR1. EGR1 directly binds to the GPX4 promoter region, thereby inhibiting GPX4 transcription. As a result, mesenchymal stromal cells become more sensitive to ferroptosis, which provides new insights for the treatment of liver diseases [93].

#### Histone acetylation regulates ferroptosis

Histone acetylation disrupts histone binding to DNA, leading to nucleosome deaggregation and gene transcriptional activation. Histone acetylation depends on Bromodomain-containing protein, BRD family, Histone acetyltransferases (HATs) and Histone deacetylases (HDACs) [94]. The BRD family can recognize acetylation markers, and the BRD4 inhibitor JQ1 can enhance the expression of HDAC called SIRT1, inducing ferroptosis by reducing H3K27ac levels upstream of BRD4 and affecting the recognition of acetylation sites on GPX4 and SLC7A11 genomic proteins [95]. Studies have shown that aberrations in HDAC3 can lead to the suppression of GPX4, thereby driving renal ferroptosis and the progression of acute kidney injury and chronic kidney disease. This inhibitory effect on GPX4 May be related to the action of HDAC3 in combination with Kruppel-like factor 5 [96].

Histone acetylation by HATs is linked to transcriptional activation [97]. NRF2 recruits P300, CBP and P300/CBP associated factors (PCAF) to increase H3K9ac levels of NRF2 and regulate ferroptosis in renal tubulointerstitial fibrosis [98]. The lysine acetyltransferase 5 (KAT5) inhibitor ketamine reduces H3K27ac levels in the GPX4 promoter region, which boosts ferroptosis from breast cancer [99]. Hepatocyte nuclear factor 4 alpha (HNF4A) and HIC ZBTB transcriptional repressor 1 (HIC ZBTB transcriptional repressor 1) in liver cancer HIC1) binds competitively to KAT2B, inhibiting the production of GSH and promoting ferroptosis [100].

Similar to HAT inhibitors, HDACS-mediated histone deacetylation also exhibits transcriptional inhibition, which triggers the induction of ferroptosis by inhibiting EMT markers in cancer cells [101]. The effect of HDAC in the tumor microenvironment is reversed by an inhibitor, and the HDAC inhibitor BEBT-908 acetylates p53 to promote ferroptosis signaling [102]. However, the regulation of ferroptosis by different cells after the same HDAC inhibitor intervention may be opposite [103]. In neurons and cancer cells with a similar mechanism of Erastin induced ferroptosis, Class I HDAC inhibitors enhance the ferroptosis of cancer cells while protecting neurons from ferroptosis [104]. This specific effect may be attributed to variations in the expression of HDAC between cancer cells and primary neurons [105].

#### Histone ubiquitination regulates ferroptosis

Histone ubiquitination related to ferroptosis in cancer cells, particularly involving Histone 2A ubiquitination (H2Aub) and Histone 2B ubiquitination (H2Bub). Both are associated with SLC7A11 expression [106]. The reduction of the tumor suppressor protein BAP1 and the ubiquitin ligase PRC1 of H2Aub can increase H2Aub uptake of SLC7A11 promoter [107]. The regulation of H2Aub by PRC1 and BAP1 leads to contrasting outcomes, yet both suppress the expression of SLC7A11 [108]. Furthermore, p53 promotes the recruitment of ubiquitin specific peptidase 7 (USP7), resulting in diminished H2Bub levels at the regulatory region of SLC7A11, thus lowering SLC7A11 expression independently of p53 as a transcription factor [106].

**Figure 2.**
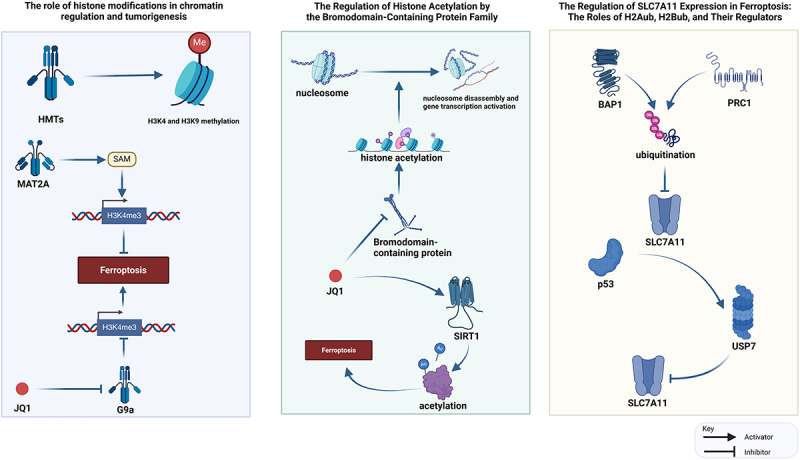
The multifaceted role of histone modifications in regulating cellular ferroptosis.

Histone modification can regulate cell susceptibility to ferroptosis by affecting the expression of associated genes and metabolic pathways of ferroptosis. However, the influence of histone modification on ferroptosis is context-dependent; different modification patterns may either counteract or synergistically affect iron-dead cells [109]. Further exploration is essential to comprehensively understand how histone modification regulates ferroptosis (Figure 2).

### DNA methylation regulates ferroptosis

DNA methylation is a prevalent epigenetic modification in eukaryotic cells, typically utilizing S-adenosyl methionine (SAM) as a methyl donor, predominantly catalyzed by DNA methyltransferases (DNMTs) such as DNMT1, DNMT3A, and DNMT3B. This process is often inversely linked to gene expression levels [110,111]. In the context of ferroptosis regulation, DNA methylation influences lipid metabolism. In mesenchymal gastric cancer cells (GCs), Elongated long-chain fatty acid protein 5 (ELOVL5) and fatty acid desaturase 1 (FADS1) promote unsaturated fatty acid synthesis, but in enteric GC, these genes are suppressed by DNA methylation, rendering the cells resistant to ferroptosis [112]. Lymphospecific helicase (LSH) in lung cancer activates metabolic genes by modifying DNA methylation through WD repeat domain 76 (WDR76) to reduce lipid ROS levels and inhibit ferroptosis [113], where LSH action is antagonized by DDB1- and CUL4-associated factor 8 (DCAF8) [114].

**Figure 3.**
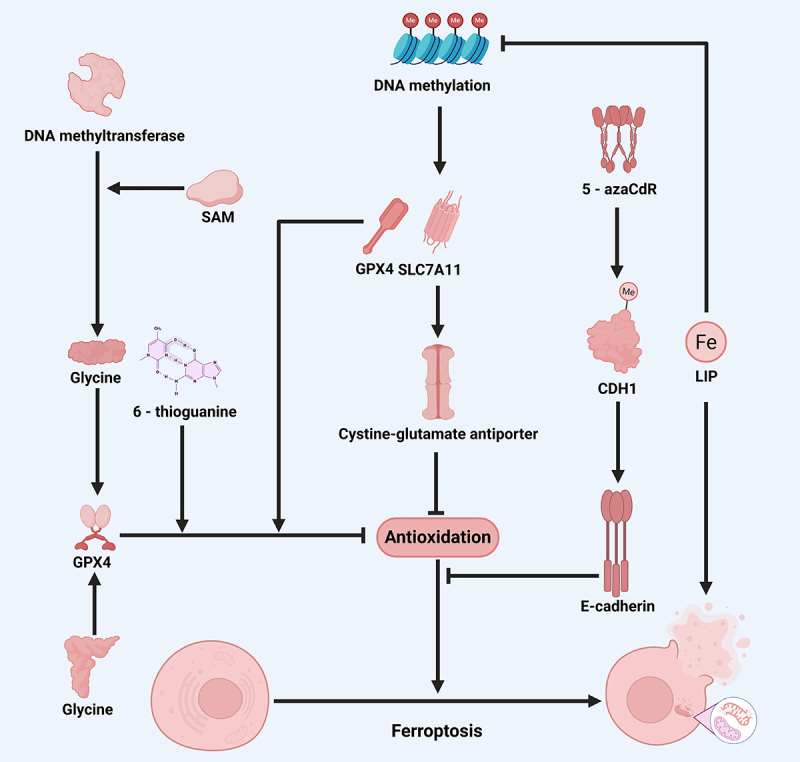
The key role of DNA methylation in the regulation of ferroptosis.

DNA methylation can directly regulate the expression of ferroptosis related molecules such as GPX4 and SLC7A11. Glycine enhances SAM-mediated Gpx4 promoter methylation catalyzed by DNMT1, DNMT3A and DNMT3B to induce ferroptosis [115,116]. The DNMT1 inhibitor 6-thioguanine is an inducer of ferroptosis in gastric cancer, which may be related to its indirect inactivation system Xc−. In addition, DNA methylation involves in the regulation of intercellular interactions in ferroptosis. DNMT1 inhibitor 5-azaCdR decreases cadherin-1 (CDH1) methylation levels, increases e-cadherin expression, and decreases ferroptosis sensitivity in head and neck cancers. Thus, DNA methylation actively regulates ferroptosis in tumor [117]. Notably, oxidative stress and iron metabolism directly affect DNA methylation levels [118]. Chronic iron exposure increases LIP levels in colon cells and promotes ferroptosis while triggering demethylation of NRF2 targets such as NOQ1 and GPX2, avoiding the occurrence of ferroptosis [119,120]. In essence, DNA methylation plays a pivotal role in ferroptosis regulation through various mechanisms, including lipid metabolism and cellular interactions [121] (Figure 3).

### RNA methylation regulates ferroptosis

RNA methylation stands as a prominent area in epigenetic investigations, constituting over 60% of all RNA modifications, with RNA m6A emerging as the most prevalent mRNA post-transcriptional alteration [122]. The biological roles of RNA m6A modification are guided by ‘readers,’ ‘writers,’ and ‘erasers.’ Writers such as METTL3, METTL14, WTAP, and KIAA1429 primarily orchestrate RNA methylation, while erasers like ALKBH5 and FTO play pivotal roles in m6A demethylation. Readers encompass RNA m6A binding proteins, notably the YTH domain protein family and the HNRNP family, which identify mRNAs featuring m6A markings [123]. Research indicates that the YTH domain of m6A (YTHDC2) can bind to SLC7A11 mRNA, facilitating its degradation [124]. In non-small cell lung cancer, FSP1 (ferroptosis resistance gene) has an mRNA carrying five m6A sites and is upregulated by ῠ3 binding. Notably, in type A AD patients, the METTL3 protein level inversely correlates with FSP1 expression, hindering SLC7A11 expression and fostering ferroptosis in human aortic smooth muscle cells. Conversely, in hepatoblastoma and lung adenocarcinoma, METTL3 bolsters SLC7A11 mRNA stability, impeding ferroptosis [125]. This may depend on how readers express it.

YTHDF1 incentivizes mRNA translation. In liver cancer, YTHDF1 recognizes m6A markers on SLC7A11 mRNA, intensifying ferroptosis inhibition, while in liver stellate cells, YTHDF1 identifies m6A markers on BECN1 mRNA, amplifying ferritin phagocytosis and triggering ferroptosis [126]. In contrast, YTHDF2 induces mRNA degradation. Studies coupling YTHDF2 with METTL14 unveil that YTHDF2-mediated degradation of SLC7A11 mRNA necessitates METTL14-mediated RNA m6A modification in hepatocellular carcinoma [125,127,128]. In lung adenocarcinoma, YTHDC2 targets SLC3A2 and SLC7A11 in an RNA m6A-dependent manner, acting as an endogenous ferroptosis inducer [129].

**Figure 4.**
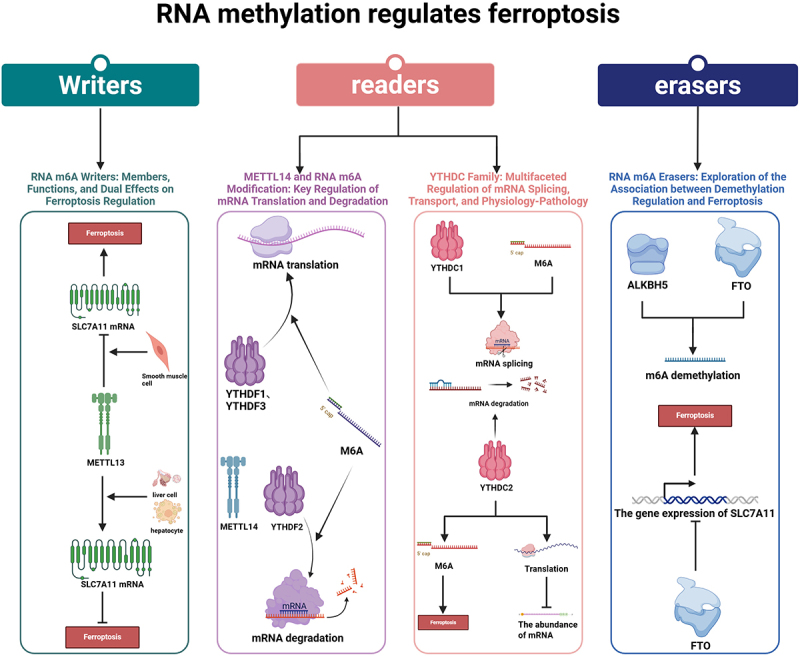
RNA methylation and its role in the regulation of ferroptosis.

Erasers also take part in the regulation of ferroptosis. In hypopharyngeal squamous cell carcinoma, ALKBH5 targets RNA m6A residues in the NRF2 transcript 3 ‘UTR to inhibit transcription and promote ferroptosis [130]. The downregulation of ALKBH5 by black phosphorus quantum dots heightens overall RNA m6A levels in lung cells, leading to lipid peroxidation, iron accumulation, and mitochondrial dysfunction. FTO down-regulates SLC7A11 expression and induces ferroptosis in thyroid carcinomates [131]. Presently, RNA m6A modification research primarily focuses on readers and writers, with less attention on erasers, warranting further exploration by the academic community. Moreover, RNA modifications extend beyond m6A labeling, with common methods like 2’-O-methylation (Nm) and pseudouracil (ψ) holding promise as future research focal points (Figure 4).

### Non-coding RNA regulates ferroptosis

In recent years, an increasing number of Noncoding RNAs (ncRNAs) with biological functions have been discovered, which can be divided into constitutive ncRNAs and regulatory ncRNAs. Constitutive ncRNAs, reminiscent of housekeeping genes, play roles in translation and splicing, including ribosomal RNA (rRNA), transfer RNA (tRNA), and small nuclear RNA (snRNA). In contrast, regulatory ncRNAs are primarily involved in transcription and post-transcriptional modifications.

MicroRNAs (miRNAs) are a class of non-coding single-stranded RNA molecules with a length of about 22 nucleotides, encoded by endogenous genes, regulating gene expression at the mRNA level [132]. They participate in epigenetic regulation mainly by binding to the target sequence 3 ‘UTR to inhibit mRNA translation or promote mRNA degradation [133]. In the classical signaling pathway, the down-regulation of SLC7A11 expression by miR-5096, miR-375 and miR-378a-3p induces ferroptosis, while the inhibition of GPX4 expression by miR-15a-5p, miR-324-3p, miR-182-5p and miR-541-3p promotes ferroptosis [134–139]. Studies have shown that miR-302a-3p and miR-335 target transferrin and ferritin respectively and promote ferroptosis by regulating iron metabolism [140]; miR-30e-5p targets specific protein 1 (SP1) and inhibits AMPK pathway to induce cell ferroptosis; miR-214-3p promotes ferroptosis by targeting ATF4. ATF4 is a key gene in ER stress [141]. Endoplasmic reticulum stress plays a dual role in ferroptosis, with ATF4 inhibiting ferroptosis by upregulating SLC7A11 in human glioma cells, while ATF4 upregulating ChaC glutathione-specific glutamylcyclotransferase 1 (CHAC1) expression in breast cancer, promoting ferroptosis induced by cystine starvation [142]. Studies have suggested that miRNA regulating genes related to ferroptosis not only acts on one gene: miR-7-5p can simultaneously up-regulate the expression of ferritin, down-regulate the expression of ALOX12, and coordinate to reduce the level of LiperFluo to inhibit ferroptosis. miRNAs may also coordinate with other epigenetic mechanisms to dynamically regulate cellular ferroptosis.

Long noncoding RNAs (lncRNAs) are ncRNAs with a length greater than 200 nucleotides [143]. Its abnormal expression is closely related to tumors, degenerative diseases, ischemic injury, etc., and is widely involved in the regulation of ferroptosis [144,145]. LncRNAs predominantly regulate ferroptosis through post-transcriptional processes. For instance, lncRNA PVT1 regulates SLC7A11 by activating p53 expression via miR-214 [146]. OIP5-AS1 and SLC16A1-AS1 inhibit ferroptosis by targeting miR-128-3p and up-regulating SLC7A11 expression [147]. lncRNA ZFAS1 inhibits the expression of miR-150-5p and targets the glutamine absorption metabolic regulator SLC38A1 to induce ferroptosis [148]. LncRNAs NEAT1 and PR11–89 modulate cellular iron concentrations to regulate ferroptosis; the former increases and the latter decreases iron levels, with NEAT1 upregulating TFR and GOT1 via miR-9-5p, while PR11–89 upregulates PROM2 via miR-129-5p [149]. LncRNA MT1DP targets miR-365a-3p to downregulate NRF2 expression, increasing cellular sensitivity to ferroptosis [150].

lncRNA can also bind to mRNA to regulate the translation process. For example, lncRNA GABPB1-AS1 directly inhibits GABPB1 mRNA translation, down-regulating peroxidoredoxin 5 expression to induce ferroptosis [151]. Some lncRNAs regulate mRNA translation through lncRNA-protein complexes. LncRNA 00925 binds to Pumilio RNA binding family member 2 (Pum2) protein, resulting in Prdx6mRNA degradation, while lncRNA ASMTcl-AS1 recruits U2AF2, stabilizing SAT1 mRNA structure to induce ferroptosis. Beyond post-transcriptional regulation, lncRNAs also modulate gene transcription directly or indirectly [152]. lncRNA Meg3 binds directly to p53 to induce ferroptosis through the p53-GPX4 axis, while cytoplasmic lncRNA P53RRA collaborates with rasGTpase activating protein-binding protein 1 (GABP1) to activate p53 gene [153]. lncRNA LINC00618 promotes ferroptosis in an apoptosis-dependent manner, inhibiting SLC7A11 expression by attenuating LSH, so as to induce the transcription of SLC7A11 after recruitment to the SLC7A11 promoter.

**Figure 5.**
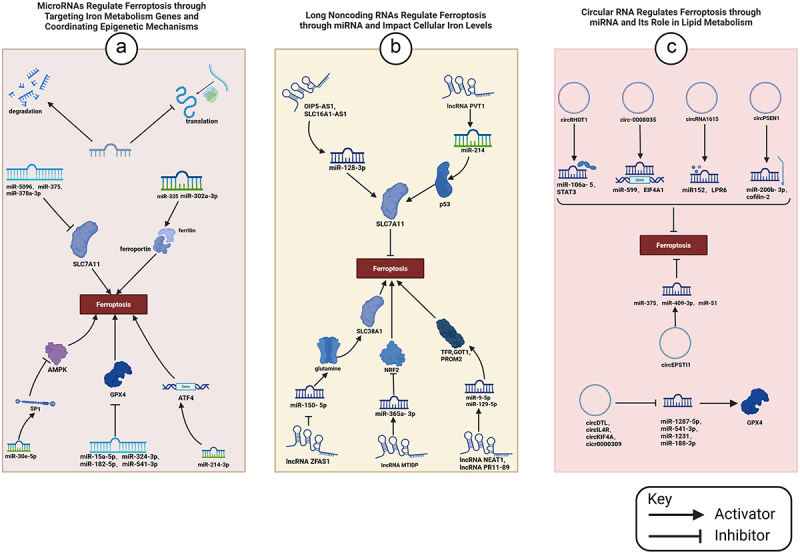
The role of noncoding RNAs in the regulation of ferroptosis.

Circular noncoding RNAs (circRNAs) are continuous rings of single-stranded RNA without A 5 ‘cap and 3’ end poly (A) tail, which eliminate inhibition of target genes by binding miRNA [154]. They are the focus of research in the field of ncRNA [155]. Depending on the targeted miRNA, the regulations of circRNA on ferroptosis vary. Several tumor-associated circRNAs influence ferroptosis through a molecular regulatory network involving circular RNA/miRNA interactions [156]. CircRHOT1, circ -0,008,035, circRNA1615 and circPSEN1 pass miR-106a-5/STAT3, miR-599/EIF4A1, miR152/LPR6 and miR-200b-3p/cofilin-2 axis respectively to inhibit ferroptosis [157]. In lipid metabolism, circPtpn14 inhibits ferroptosis by targeting miR-351-5p. circDTL, circIL4R, circKIF4A and cicr0000309 bind to inhibit miR-1287-5p, miR-541-3p, miR-1231 and miR-188-3p respectively to increase GPX4 expression [158]. A single circular RNA can carry multiple miRNA binding sites, for circEPSTI1 inhibits ferroptosis by simultaneously binding miR-375, miR-409-3p, and miR-51 (Figure 5).

## The application prospect of targeted ferroptosis epigenetic regulators in tumor therapy

Epigenetic alterations in cancer are reversible, presenting an appealing avenue for cancer therapy. Targeting ferroptosis through epigenetic modulation emerges as a promising strategy in cancer treatment, with relevant research integrated into anti-cancer therapeutics.

The BRD4 inhibitor JQ1 can trigger ferroptosis in apoptosis-resistant cancer cells, augmenting the anticancer efficacy with ferroptosis inducers. Mechanistically, JQ1 suppresses the expression of histone methyltransferase G9a by inhibiting BRD4, downregulating key ferroptosis-associated genes SLC7A11, SLC3A2, and GPX4, while enhancing histone deacetylase SIRT1 expression. In an IVDD mouse model, homocysteine, involved in homomethylation, boosts GPX4 methylation, instigating ferroptosis in nucleus pulposus cells [159]. The class I HDAC inhibitor vorinostat can enhance ferroptosis in small cell lung cancer by regulating histone or non-histone acetylation [160]. Controlling m6A-based transcriptomics can destroy the redox balance and induce ferroptosis in leukemia cells. GNPa-CSP12 functionalized gold nanorods can eliminate endogenous Fe^2^-dependent m6A demethylase activity, and its down-regulation is closely related to glycolysis, hypoxia and immune checkpoint pathway-related genes [161]. Tiliroside is a potential natural anti-cancer product that can target TBK1 to reduce the phosphorylation level of serine 349 on sequestosome-1 (p62), thereby decreasing the affinity of p62 for Kelch-like ECH-associated protein 1 (Keap1) and promoting Keap1-mediated ubiquitination and degradation of NRF2, which induces ferroptosis in hepatocellular carcinoma [162]. Corosolic acid can reduce the ubiquitination level of the E3 ubiquitin ligase MDM2 associated with GSS through HERPUD1, thereby promoting the ubiquitination of GSS and inhibiting GSH synthesis, which in turn enhances the sensitivity of liver cancer cells to ferroptosis [163]. QD394 is a quinazolinedione ROS inducer which can induce ferroptosis by inhibiting STAT3 phosphorylation, thereby inhibiting GPX4 expression in pancreatic cancer [164]. Eriodictyol induces ferroptosis by downregulating NRF2 phosphorylation, thereby decreasing the protein levels of SLC7A11 and GPX4 in ovarian cancer [165]. The nexus of epigenetic drug-mediated ferroptosis regulation with conventional chemotherapy, targeted therapy, immunotherapy, and radiotherapy presents substantial research potential and application prospects in cancer care (Table 3). However, the application of epigenetic regulation of ferroptosis in cancer treatment still requires a great deal of further research. The safety of regulating ferroptosis to achieve cancer treatment effects needs further investigation, as ferroptosis occurs in both normal and tumor cells [2]. Moreover, whether epigenetic mechanisms affect multiple ferroptosis-related genes and how these different epigenetic mechanisms interact with diverse signaling pathways to determine the cellular response to ferroptosis stimuli remain unclear. Further research is needed to elucidate these questions [167].

**Table 3.** Targeting ferroptosis epigenetic regulator drugs in tumor therapy.

Name of drug	Target of action	Mechanism of action	Types of diseases addressed	References
JQ1	BRD4	Reduced G9a expression by inhibiting BRD4, down-regulated the expression of ferroptosis-related genes SLC7A11, SLC3A2 and GPX4, and enhanced the expression of SIRT1.	Cancer	Alborzinia et al.[166]
Homocysteine	GPX4	Enhanced GPX4 methylation leads to ferroptosis in myeloid cells.	Cancer	Cai et al. [144]
Vorinostat	HDAC	Regulation of histone or non-histone acetylation enhances ferroptosis in small cell lung cancer.	Small-cell lung cancer	Yao et al. [145]
Gold nanorod	m6A	Elimination of endogenous Fe^2+^-dependent m6A demethylase activity, which is downregulated in close association with genes related to glycolysis, hypoxia, and the immune checkpoint pathway.	Leukaemia	Lu et al. [146]
Tiliroside	TBK1	Targeted TBK1 to induce ferroptosis in cancer cells.	Hepatocellular carcinoma	Yang et al. [162]
Corosolic acid	HERPUD1	Decreased the cellular GSH level and caused liver cancer cells to become more sensitive to ferroptosis.	Hepatocellular carcinoma	Peng et al. [163]
QD394	STAT3	Inhibited STAT3 phosphorylation, thereby inhibited GPX4 expression.	Pancreatic cancer	Hu et al. [164]
Eriodictyol	NRF2	Downregulated NRF2 phosphorylation, thereby decreased the protein levels of SLC7A11 and GPX4.	Ovarian cancer	Wang et al. [165]

## Conclusion and prospect

Epigenetics intricately influences ferroptosis in tumor cells, modulating ferroptosis-related genes and pathways without altering tumor gene expression levels. Current investigations on the epigenetic control of ferroptosis in tumor cells are extensive. Given the reversible nature of epigenetics, its modulation of tumor cell ferroptosis stands as a promising therapeutic target with bright application potential. However, we still need more research to confirm the efficacy of epigenetic drugs in ferroptosis regulation therapy. Hence, further exploration of the interplay between diverse epigenetic regulations and tumor ferroptosis, alongside the development of tumor-targeted drugs for distinct targets, in conjunction with conventional treatments, offers novel insights for cancer treatment.

## Data Availability

No data was used for the research described in the review.
